# The Influence of Household- and Community-Level Sanitation and Fecal Sludge Management on Urban Fecal Contamination in Households and Drains and Enteric Infection in Children

**DOI:** 10.4269/ajtmh.16-0170

**Published:** 2017-06-07

**Authors:** David Berendes, Amy Kirby, Julie A. Clennon, Suraja Raj, Habib Yakubu, Juan Leon, Katharine Robb, Arun Kartikeyan, Priya Hemavathy, Annai Gunasekaran, Ben Ghale, J. Senthil Kumar, Venkata Raghava Mohan, Gagandeep Kang, Christine Moe

**Affiliations:** 1 Department of Environmental Engineering, School of Civil and Environmental Engineering, Georgia Institute of Technology, Atlanta, Georgia; 2 Center for Global Safe Water, Sanitation, and Hygiene, Rollins School of Public Health, Atlanta, Georgia; 3 Hubert Department of Global Health, Rollins School of Public Health, Emory University, Atlanta, Georgia; 4 Department of Biostatistics and Bioinformatics, Rollins School of Public Health, Emory University, Atlanta, Georgia; 5 Wellcome Research Laboratory, Christian Medical College, Vellore, India; 6 Department of Community Health, Christian Medical College, Vellore, India

## Abstract

Urban sanitation necessitates management of fecal sludge inside and outside the household. This study examined associations between household sanitation, fecal contamination, and enteric infection in two low-income neighborhoods in Vellore, India. Surveys and spatial analysis assessed the presence and clustering of toilets and fecal sludge management (FSM) practices in 200 households. Fecal contamination was measured in environmental samples from 50 households and household drains. Enteric infection was assessed from stool specimens from children under 5 years of age in these households. The two neighborhoods differed significantly in toilet coverage (78% versus 33%) and spatial clustering. Overall, 49% of toilets discharged directly into open drains (“poor FSM”). Children in households with poor FSM had 3.78 times higher prevalence of enteric infection when compared with children in other households, even those without toilets. In the neighborhood with high coverage of household toilets, children in households with poor FSM had 10 times higher prevalence of enteric infection than other children in the neighborhood and drains in poor FSM clusters who had significantly higher concentrations of genogroup II norovirus. Conversely, children in households with a toilet that contained excreta in a tank onsite had 55% lower prevalence of enteric infection compared with the rest of the study area. Notably, households with a toilet in the neighborhood with low toilet coverage had more fecal contamination on floors where children played compared with those without a toilet. Overall, both toilet coverage levels and FSM were associated with environmental fecal contamination and, subsequently, enteric infection prevalence in this urban setting.

## Introduction

Poor water, sanitation, and hygiene are associated with multiple adverse health and developmental outcomes.^1^,^2^ Following the Millennium Development Goals, the focus of the sanitation sector has been in rural areas, where 70% of those without access to improved sanitation live.^3^ However, the need for sanitation solutions in poor urban neighborhoods and informal settlements is a growing concern, as the world's population has shifted to being predominantly urban within the last decade.^4^,^5^ By 2050, the urban population is estimated to almost double, from 3.3 to 6.3 billion, providing a new challenge for sanitation implementers and policymakers.^4^ Within poor, dense urban neighborhoods with little sanitation infrastructure, frequent person-to-person contact and poor environmental conditions facilitate transmission of fecal–oral infections, yielding frequent diarrhea in young children.^4^,^6^–^11^

Despite links between poor sanitation and health, evidence of effective sanitation in urban settings remains weak. Recent meta-analyses have identified few interventions in urban neighborhoods and limited evidence, overall, of the positive effects of sanitation on diarrheal disease.^12^,^13^ Among other limitations, the authors highlight bias in outcome measures (self-reported diarrhea) and poor mechanistic evidence of changes in more proximal exposures, such as fecal contamination, with concurrent changes in sanitation coverage.^12^ While several quantitative microbial risk assessments (QMRAs) have modeled environmental transmission pathways for exposure to fecal contamination and risk of enteric infection, few studies have measured associations between microbial indicators or pathogens in the household or public environments and type or coverage of sanitation.^14^,^15^ Because effective sanitation is expected to decrease enteric infection risk through safe containment of excreta, it is important to examine changes in environmental fecal contamination as an intermediate outcome.^16^

Systems-level approaches to urban sanitation, where containment of excreta requires more than a household toilet alone, have not been well examined.^5^ When compared with rural settings, urban sanitation presents complex challenges, in particular the spillover of fecal contamination from private to public domains and vice versa. Preventing this spillover requires consideration of the entire sanitation chain to ensure safe containment, transport, treatment, and ultimately disposal or reuse of excreta.^17^ Components of the sanitation chain start with the user interface (household toilet), but also include transport (e.g., sewerage or onsite containment followed by emptying and trucking of fecal sludge) and eventual treatment of the fecal sludge. These components beyond the toilet are encompassed by the current focus on “fecal sludge management” (FSM).^18^ To date, associations between urban FSM, fecal contamination, and adverse health outcomes have only been evaluated for sewerage interventions, which were associated with reduced diarrhea incidence.^19^ Because sewerage may not be feasible in some urban settings and because many current sewer and open drain connections do not result in treatment of the fecal sludge, it is important to examine the effects of other FSM models, including onsite containment, on health outcomes.^6^

In addition to the linear sanitation chain, studies must also consider the spatial heterogeneity of urban sanitation coverage. While the effect of toilets on environmental fecal contamination and enteric infection is often measured at the household level, sanitation may have community-level benefits in areas of high sanitation coverage, even for residents without a toilet.^20^–^22^ Because of the interconnectedness of public and private urban environments, there is a growing need to examine how the concentrations of fecal contamination in the environment vary with the underlying spatial distribution and clustering of sanitation in the community.^23^

While management of fecal sludge in low-income, urban areas is generally poor, the associations between FSM and fecal contamination or enteric infection within that environment have not been examined.^7^ There is a need to understand how fecal contamination and enteric infection vary with toilets and FSM in the urban environment. This study examines the associations between household sanitation, including toilets, their associated FSM, and their spatial clustering, and fecal contamination within the household and nearby drains, as well as pediatric enteric infection. By assessing proximal exposure outcomes and more distal infection outcomes in two different urban neighborhood environments, this work will contribute to understanding how the urban environment affects the success of sanitation interventions.

## Materials and Methods

### Data source.

This study was conducted as a sub-study of a SaniPath Exposure Assessment Tool^24^ deployment in two low-income urban neighborhoods in Vellore, India. Data were collected in February–March and September 2014.

### Study site.

The study site consisted of two neighborhoods in Vellore, Chinnallapuram and Old Town, which were chosen because of their low socioeconomic status, poor sanitation, and long-standing relationship with the Christian Medical College (CMC), which provided access to spatial and demographic data from previous studies.^25^,^26^ The Old Town neighborhood is the site for the Interactions of Malnutrition and Enteric Infections: Consequences for Child Health and Development (MAL-ED) study in Vellore.^25^ Of the seven contiguous sub-neighborhood areas selected for the MAL-ED study, five were selected for the SaniPath Tool deployment.

The Chinnallapuram study area is a 0.41 km^2^ semi-urban neighborhood with a reported population density of 30,520/km^2^.^27^ Old Town is a 0.33 km^2^ urban neighborhood with an estimated population density of 41,977/km^2^.^25^ The study area within Old Town was approximately 0.18 km^2^. Vellore is subject to two monsoon seasons (a southwest monsoon from June to September and a northeast monsoon from October to December), with the remaining January to May period as a dry season.^25^

Among the study population, houses had floors made of cement or concrete (84%), ceramic tiles (10%), or earth or mud (6%). Walls were mostly cement or concrete also (81%), though 15% had mud walls.^25^ Households were mostly Hindu (59.9%) or Muslim (33.7%) while few were Christians (6.4%, data from a local census maintained by the Department of Gastrointestinal Sciences at CMC). Sanitation consisted of pour-flush toilets with slabs within the household (household toilets), public toilets, and open defecation. Water sources were predominantly municipal taps, providing intermittent water supply for a few days each week, thus water was stored at the home as well. Water treatment (boiling) was practiced by some of the population and was recorded as part of the CMC hygiene survey (below and Supplemental Information).

In each neighborhood, environmental samples and stool specimens were collected from 25 households selected 1) from neighborhood sampling frames from previous CMC studies and 2) based on the score from a hygiene survey developed by CMC, previously validated, and implemented 1 month before SaniPath data collection.^28^,^34^,^35^ Briefly, this survey assessed 18 household hygiene characteristics and behaviors related to water collection, infant cleaning and feeding practices, and defecation, using Yes (equal to 1) or No (equal to 0) responses to create an additive household hygiene score. Scores less than or equal to 9 were classified as “poor” hygiene, whereas scores greater than 9 were classified as “good” hygiene. The hygiene survey was administered to all households in each study neighborhood before the study's commencement. To ensure variation in general household-level hygiene practices, 13–14 poor hygiene (those with the lowest scores in the survey) and 11–12 good hygiene households (those with the highest scores in the survey) were selected randomly in each neighborhood. Further detail, including the survey instrument, can be found in the Supplemental Information.

### Ethical approval.

Approval was obtained from the Emory University Institutional Review Board (IRB) and the CMC IRB. Informed consent was obtained before sample collection and survey administration at each household.

### Environmental and stool sample collection, analysis, and processing.

Environmental samples—including hand rinses from children less than 5 years of age, rinses of a sentinel object, swabs of household floors, and 500 mL of drain water—and stool specimens from children under 5 years of age were collected in March 2014. After collection, samples and specimens were stored on ice for up to 4 hours until arrival at the laboratory, where they were refrigerated at 4°C. Environmental samples were analyzed for *Escherichia coli* within 6 hours of receipt by membrane filtration and plating on m-ColiBlue24^®^ Medium (Hach Company, Loveland, CO) according to the U.S. Environmental Protection Agency method 1604.^36^ Enteroaggregative *E. coli* (EAEC) and genogroup I and II (GI and GII) norovirus were assessed by quantitative polymerase chain reaction (PCR) and reverse transcription PCR (RT-PCR). *Escherichia coli* was chosen as an indicator of fecal contamination, whereas EAEC and norovirus were chosen based on their high prevalence in children of the Vellore field site for the MAL-ED study.^37^,^38^ Further, both are predominantly human-specific infections.^39^

Stool specimens were analyzed for enteropathogens using the MAL-ED study protocols, with the exception of *Campylobacter* spp., which was assessed by PCR.^29^,^30^,^38^ Further information on pathogens analyzed is provided in the Supplemental Information.

#### Hand rinses.

At each household, a hand rinse sample was collected from the child under 5 years of age who was previously enrolled in other CMC studies. The child's right hand was inserted into a sterile 2-L Whirl-Pak bag (Nasco, Fort Atkinson, WI) containing 500 mL of sterile phosphate-buffered saline (PBS) solution. The staff massaged the fingers and palm for 30 seconds in the PBS, then the child removed their right hand, inserted their left hand, and the massage procedure was repeated. At the laboratory, the sample was diluted 1:10^0^, 1:10^1^, and 1:10^2^ in sterile PBS before membrane filtration. Before PCR, 200 mL of the original hand rinse sample was precipitated with 12% polyethylene glycol (PEG) 8000, centrifuged for 20 minutes at 6,000 rpm, and suspended in 5 mL sterile water, of which 1.5 mL was further concentrated by precipitation with 12% PEG 8000 before nucleic acid extraction.^40^

#### Sentinel objects.

At each household, a child's toy or feeding spoon, volunteered by the mother, was used as the “sentinel object.” The object was inserted into a sterile 2-L Whirl-Pak bag containing 500 mL of sterile PBS, massaged from the outside of the bag for 1 minute, and subsequently removed and returned to the family. Rinses from sentinel objects were processed identically to hand rinse samples for membrane filtration and PCR.

#### Household floor swabs.

At each household, a composite household floor swab was collected using EnviroMax Plus Sterile Environmental Swabs (Puritan Medical Products, Guilford, ME). The child's play area was identified by the mother, after which the field staff used a 24 × 16-cm framing square to outline a 25 cm^2^ area in each of four corners and the center of the play area and swabbed back and forth across those sections of the floor. Two swabs were used to cover the entire area and subsequently combined into a single sample covering a total surface area of 125 cm^2^. Each swab was eluted in 7 mL of PBS solution in a sterile container, and the eluates from both swabs were combined for an approximate sample volume of 14 mL. From this volume, dilutions of 1:10^0^, 1:10^1^, and 1:10^2^ were made and membrane filtered. Nucleic acids were extracted from 1.5 mL of swab eluate after one round of PEG precipitation.

#### Drain water.

A sample of drain water was collected from the drain directly in front of the household, regardless of the discharge location of the household toilet (if applicable). A sterile bailer or stainless steel ladle was used to collect approximately 500 mL of drain water into a sterile 2-L Whirl-Pak bag, taking care not to disturb sediment on the bottom or nearby trash. Drain samples were diluted 1:10^1^, 1:10^2^, and 1:10^3^ in sterile PBS at the laboratory before membrane filtration. DNA and RNA were extracted from 1.5 mL samples of the original sample before PCR analysis.

Because almost all drain water samples collected during the initial (February–March) sampling period had *E. coli* colony numbers above the countable range on the filter membrane, 10 of the original 25 households in each neighborhood were resampled spatially at random in September 2014, per the original sampling protocol. These samples were analyzed by membrane filtration for *E. coli* after 1:10^4^–1:10^6^ dilutions.

#### Stool specimens.

A stool specimen was collected from each child under 5 years of age in the 50 study households. Further detail on the analysis methods is given in the Supplemental Information.

#### Quantitative real-time PCR.

Further detail on probes, primers, and standard curves used in quantitative PCR analyses can be found in the Supplemental Information. All samples were tested using the Qiagen QuantiFast Pathogen + IC Kit (Qiagen Sciences, Germantown, MD) (PCR for EAEC and RT-PCR for norovirus) for initial screening and assessment of potential PCR inhibition. Any samples that were positive (at least one well with a cycle threshold (C_t_) value less than 45) or inhibited were quantified using the OneStep PCR (EAEC) or RT-PCR (norovirus) kit (Qiagen) and a standard curve. Positive and negative controls for EAEC or norovirus were included with every PCR run.

Samples tested for GI or GII norovirus using the OneStep Kit and classified as positive (both wells had C_t_ values less than or equal to 45 and a difference of less than or equal to 4 between C_t_ values for duplicate wells) were quantified by a simple average of both wells. Due to inconsistent standard curves affecting quantification—but not detection—of EAEC, samples tested for EAEC using the OneStep kit were classified as positive or negative. Samples with no detectable EAEC or norovirus were assigned the value of the theoretical lower limit of detection for the assay (334 cell equivalents [CE] for EAEC or genome equivalent copies (GEC) for norovirus GI and GII per 100 mL (2.52 log_10_ CE or GEC/100 mL)).

### Survey data collection.

In each study neighborhood, surveys were conducted in 100 households: 25 households were those with concurrent environmental sample and stool specimen collection, whereas the remaining 75 were divided equally across sub-neighborhood areas and chosen at random within them. To be eligible, households had to have a child under 5 years of age. The target respondent for the survey was the person responsible for water, sanitation, hygiene, and food activities, generally the mother of the youngest child, or rarely, the grandmother. If the respondent was not available and the household was one of the households where environmental stool samples were to be collected, survey enumerators returned to the household at a later time, otherwise, the nearest available household was selected for survey. The household survey included questions about the household's population, presence of a toilet, and FSM practices, as well as the children's and adult's defecation practices. A Global Positioning System location was collected at each household using Garmin eTrex Venture HC devices (Garmin International Incorporated, Olathe, KS).

### Analyses.

Concentrations per pair of hands, sentinel object, and 125 cm^2^ of household floor were back-calculated using the rinse volume for these samples. All microbial concentrations were log_10_-transformed before statistical analyses. Values below the lower detection limit for membrane filtration were substituted on the log_10_ scale with the value of the lower limit of detection (1 colony-forming unit [CFU]/100 mL), accounting for sample dilution.

Statistical analyses were conducted in R version 3.2.3 (R Foundation for Statistical Computing, Vienna, Austria) using base packages and the “lme4” package for mixed-effects logistic regression.^41^–^44^ Linear regression was used to assess continuous outcomes (*E. coli* concentrations in all environmental samples and norovirus GII concentrations in drain samples) while logistic regression was used to assess binary outcomes (presence/absence of EAEC and norovirus GII in drain samples, as well as pathogen detection in stool specimens).

Binary (presence/absence) data from household surveys were evaluated for most-likely local clustering in SaTScan version 9.4 (SaTScan.com, Boston, MA) using Kulldorff's Bernoulli spatial scan, which evaluates binary outcomes in point data distributed in space to assess the degree of nonrandom clustering of “0” or “1” values.^45^ An α of 0.05 was used to determine significance in cluster analysis and regression modeling.

## Results

### Frequency and within-neighborhood spatial clustering of household sanitation.

**Figure 1.**
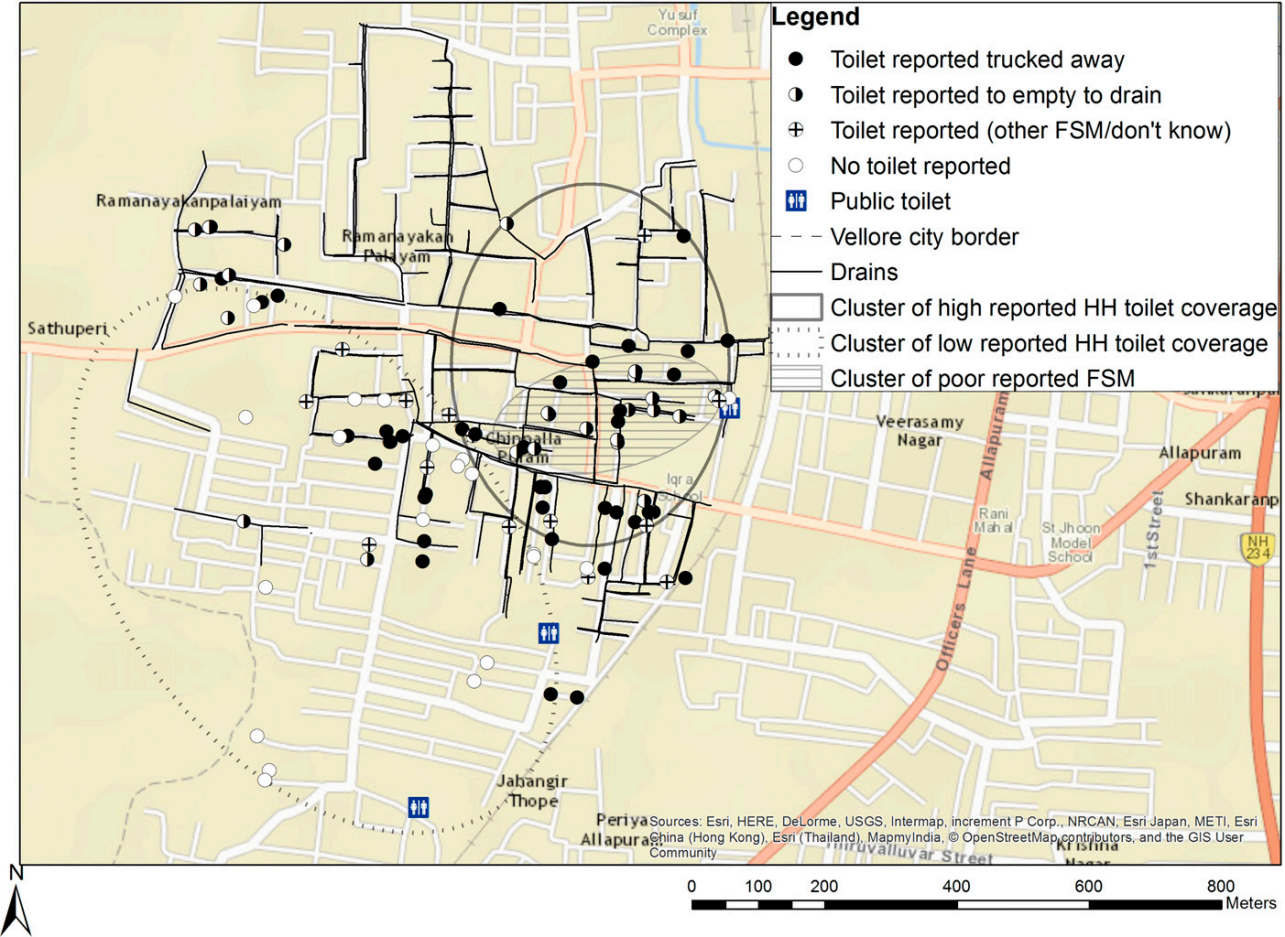
Sanitation coverage and clustering in Chinnallapuram.

To compare sanitation coverage and spatial heterogeneity within and between study neighborhoods, we assessed the frequency and type of household toilets and FSM and their most-likely clustering in Chinnallapuram and Old Town (Table 1, Figures 1

and 2

**Figure 2.**
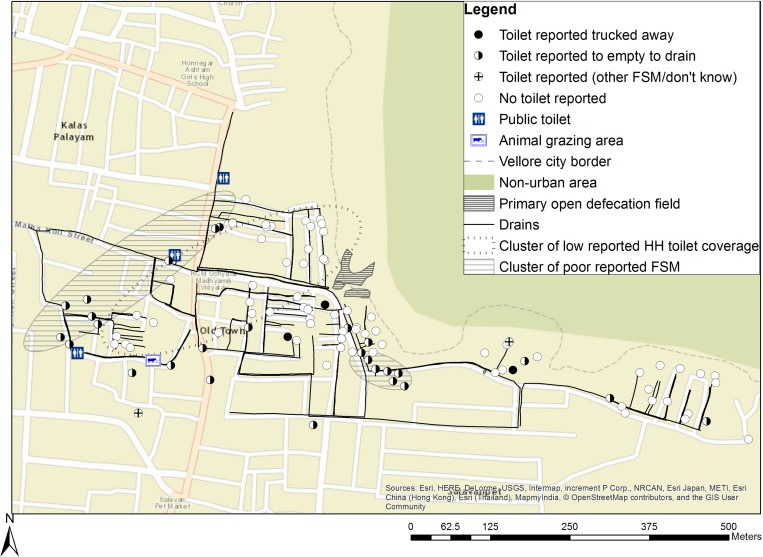
Sanitation coverage and clustering in Old Town.

). In both neighborhoods, toilets either discharged directly to an open drain (defined as “poor FSM”) or were connected to a tank under the household that contained the excreta (defined as “good FSM”). Compared with households in Chinnallapuram, those in Old Town reported a significantly lower proportion of household toilets (33% versus 78%), and more household toilets had poor FSM (82% versus 35%). Among households with toilets, reported use of toilets was high in Chinnallapuram (76/78 households) and Old Town (32/33 households, data not shown). Open defecation was more common in Old Town than in Chinnallapuram, but use of public toilets was similar (54% and 59%, *P* = 0.57). Frequent use of public toilets (more than 10 times per month) was more common in Old Town (18%) than Chinnallapuram (4%, *P* < 0.01).

Significant clustering of high coverage of household toilets was present in Chinnallapuram, but not Old Town. Significant spatial clusters of low coverage of household toilets and high coverage of household toilets with poor FSM were present in both neighborhoods (Table 1: Most likely cluster, Figures 1 and 2). Clusters of low coverage of household toilets varied in size (40 households in Chinnallapuram versus 27 households in Old Town) and coverage within the cluster (50% in Chinnallapuram versus 0% in Old Town). In Chinnallapuram, coverage of households with toilets with poor FSM was 78% in the significant cluster of poor FSM present. In Old Town, both significant clusters were small, but had 100% coverage of households with toilets with poor FSM. No significant clusters of high coverage of toilets with good FSM were detected.

### Microbiological concentrations in environmental samples.

Levels of fecal contamination in the household were characterized by examining rinses of children's hands, rinses of sentinel objects, and swabs of household floors. *Escherichia coli*, EAEC, and GI and GII norovirus levels were quantified in these samples. Distributions of sample *E. coli* concentrations were all approximately normal when log-transformed, though sentinel object rinses did have frequent left-censored values from non-detects (data not shown). *Escherichia coli* were detected in most hand rinse, sentinel object, and household swab samples (Table 2: Overall). To assess differences in fecal contamination within households by neighborhood and household hygiene practices, logistic and linear regression models were constructed for *E. coli* data (Table 2: Associations with neighborhood and hygiene status). Overall, there was no significant variation in *E. coli* detection or concentrations in samples by neighborhood or hygiene status.

EAEC was detected in 1/50 hand rinses, 0/50 sentinel objects rinses, and 1/50 floor swabs. GI norovirus was not detected in samples within the household. GII norovirus was detected in 1/50 hand rinse samples, 0/50 sentinel object rinses, and 0/50 floor swabs. These EAEC and GI and GII norovirus samples were omitted from further analysis due to low levels of detection.

Levels of fecal contamination outside the household were characterized by examining drain samples, which were analyzed for *E. coli*, EAEC, and GI and GII noroviruses. *Escherichia coli* was detected in 50/50 drain samples collected initially (February–March 2014), with concentrations above the detection limit in 49/50 samples and in 20/20 resamples (September 2014), with concentrations above the detection limit in 1/20 samples, a mean of 6.83 log_10_ CFU/100 mL (standard deviation [SD]: 0.56 log_10_ CFU/100 mL), and an approximately normal distribution when log-transformed. Because drain resampling took place during a different season than the rest of data collection, drain *E. coli* concentrations were excluded from further analyses. EAEC, GI norovirus, and GII norovirus were detected in 15/50, 1/50, and 19/50 drain samples, respectively. Mean concentrations of EAEC and GII norovirus were 2.67 log_10_ CE/100 mL (SD: 0.41 log_10_ CE/100 mL) and 3.43 log_10_ GEC/100 mL (SD: 1.41 log_10_ GEC/100 mL), respectively.

### Enteric pathogen detection in stool.

To determine the prevalence of enteric infections in children, a stool specimen from each child under 5 years of age in the 50 study households where environmental samples were collected was assayed for viral, bacterial, protozoan, and parasitic enteric pathogens. Overall, one or more enteric pathogens was detected in 51/76 stool specimens (67%). *Campylobacter* spp. was most frequently detected in stool specimens (32/76 children), but astrovirus (7/76), *Entamoeba histolytica* (1/76), *Giardia* spp. (17/76), GII norovirus (5/76), and pathogenic *E. coli* (14/76) were also detected (data not shown).

Variation in enteric pathogen prevalence by neighborhood and household hygiene status was assessed using mixed-effects logistic regression for pathogens detected frequency (> 20% of stool specimens, Table 3). The prevalence of any enteric pathogens in stool, as well as that of specific pathogens, did not vary significantly by neighborhood or household hygiene status, with the exception of *Campylobacter* spp. detection. Households with poor hygiene had significantly higher detection of *Campylobacter* spp. in stool compared with those with good hygiene (prevalence ratio: 3.42, *P* = 0.02). Because no single pathogen was associated with more than half of infections, further analyses were limited to the presence of any enteric infection (i.e., pooled pathogens) as an outcome.

### Association between household- and cluster-level toilet coverage, FSM practices, and within-household fecal contamination.

Associations between household toilet presence, FSM practices, and within-household fecal contamination were examined using multivariate linear regression (Supplemental Tables 1–3). No significant associations were observed between household- or cluster-level sanitation variables and *E. coli* concentrations on children's hands (Supplemental Table 1).

Associations between *E. coli* concentrations on floors and presence of a household toilet varied by neighborhood (Supplemental Table 2). At the household-level (ignoring spatial clustering), there was no significant difference in *E. coli* concentrations in floor swabs in households with and without a toilet in Chinnallapuram. However, in Old Town, the presence of a toilet was associated with higher *E. coli* concentrations on floors (difference of 1.15 log_10_ CFU/125 cm^2^, *P* = 0.06), compared with households without a toilet (Supplemental Table 2: Household level). At the cluster level, households in the cluster of low coverage of household toilets in Old Town (0% coverage within the cluster) had significantly lower *E. coli* concentrations on floors compared with the rest of the neighborhood (difference of −1.38 log_10_ CFU/125 cm^2^, *P* = 0.02, Supplemental Table 2: Most likely clusters). No difference in *E. coli* concentrations was observed within and outside the cluster of low coverage of household toilets in Chinnallapuram (50% coverage within the cluster).

*Escherichia coli* concentrations in rinses of sentinel objects were not significantly associated with any sanitation variables (Supplemental Table 3).

### Associations between demographics, neighborhood, and household- and cluster-level sanitation variables and fecal contamination outside the household.

To examine variation in pathogen levels in the public domain with household and neighborhood sanitation, logistic regression models for EAEC and both logistic and linear regression models for GII norovirus in drains were constructed. Though significant associations were not detected for EAEC (data not shown), large effect sizes were observed that may be worthy of future investigation. For example, EAEC was less likely to be detected in drains outside households with poor, compared with those with good, hygiene scores (odds ratio [OR]: 0.30, 95% confidence interval [CI]: 0.08–1.02, *P* = 0.06). Further, drain samples in clusters of low coverage of household toilets were less likely to be positive for EAEC (OR: 0.27, 95% CI: 0.05–1.14, *P* = 0.09), whereas those in clusters of high coverage of household toilets were more likely to be positive (OR: 5.01, 95% CI: 0.70–47.6, *P* = 0.12).

GII norovirus detection and concentrations in drains did not vary significantly by neighborhood or hygiene status (Table 4: Demographics and hygiene). At the household-level, the odds of detection and concentrations of GII norovirus in drain samples were higher for drains adjacent to households with toilets compared with those without toilets, though these differences were not significant (*P* = 0.07 and *P* = 0.10, respectively; Table 4: Sanitation (household level)). At the cluster level, GII norovirus concentrations in drains within the cluster of high toilet coverage were significantly higher (by 1.47 log_10_ GEC/100 mL, *P* = 0.01; Table 4: Sanitation (cluster level)) than in the rest of the neighborhood. Associations between clusters of poor FSM and GII norovirus concentrations in drains varied by neighborhood. In Chinnallapuram, GII norovirus concentrations were significantly higher within the cluster of high coverage of poor FSM than the rest of the neighborhood (difference in concentrations: 2.50 log_10_ GEC/100 mL, *P* = 0.02), whereas in Old Town, there was no significant association (difference in concentrations: 0.16 log_10_ GEC/100 mL, *P* = 0.07 for interaction).

### Household- and cluster-level sanitation and enteric infection in children.

Associations between household- and cluster-level sanitation and concurrent enteric infection in children (detection of any enteric pathogen in children's stool) were evaluated by mixed-effects logistic regression (Table 5). Children in households with a toilet with good FSM had 55% lower prevalence of infection, compared with children in households with toilets with poor FSM or no toilet present (*P* = 0.17; Table 5: Household level). Conversely, children in households with a toilet with poor FSM had 3.78 times higher prevalence of infection when pooled across both neighborhoods (*P* = 0.05, data not shown), though this association varied by neighborhood. In Chinnallapuram, children in households with a toilet with poor FSM had 10 times the prevalence of enteric infection of children in other households (*P* = 0.04), whereas in Old Town, no significant association was present. Similar associations were observed when separating children by households with a toilet with good FSM, those with a toilet with poor FSM, or those without a toilet. Prevalence of enteric infection did not vary significantly with cluster-level sanitation variables (Table 5: Cluster level).

## Discussion

This study examined the associations between household sanitation—including toilets, FSM, and spatial heterogeneity of sanitation coverage—and fecal contamination within the household and the local urban environment and enteric infection in young children. The results suggest that the FSM, neighborhood-level coverage, and spatial clustering of household toilets are significantly associated with household- and neighborhood-level fecal contamination and pediatric enteric infection prevalence. Enteric infection was significantly more prevalent among children in households with poor FSM, even when compared with children in households without toilets, and was least prevalent among children in households with good FSM. In areas of high coverage of household toilets, drains in clusters of poor FSM had higher concentrations of GII norovirus compared with the rest of the study area. In areas of low coverage of household toilets, the presence of a toilet was associated with higher *E. coli* concentrations on household floors.

This study is one of the first to examine urban, household toilets by both their spatial heterogeneity and associated FSM and describe associations with pediatric enteric infection. Comparison of onsite excreta containment in tanks under the household to open drainage is new to the literature, which has previously focused on sewerage.^19^,^46^,^47^ Although evidence of lower incidence of pediatric diarrhea associated with urban drainage interventions exists, our findings suggest that toilets that discharge to open drains may be potential risk factors for fecal exposure and enteric infection.^48^ Finally, this is one of the few studies to quantify fecal contamination (including fecal indicator bacteria and enteric pathogens) in both the household and public domain as an outcome and examine its associations with sanitation in low-income urban areas.

Provision of household toilets without regard for associated FSM practices may still contribute to fecal contamination in the local environment and may not reduce pediatric enteric infection when compared with the absence of a toilet, especially in dense, urban areas. High coverage of toilets discharging directly into the local environment combined with ubiquitous presence of open drains was associated with higher concentrations of human-specific pathogens, for example, GII norovirus, in drains in this study.^49^ Although structured observations from other low-income, urban areas suggest contact with drains is not common for this age group, QMRAs suggest that enteric infection may occur after a single contact.^14^,^15^,^50^,^51^ Further, children likely have frequent contact with the floors and ground both inside and outside the household, as well as contact with caregivers or other family members, all of which may provide direct or indirect exposure to feces from the outside environment.^50^,^52^ Thus, though the specific exposure pathway is unclear, there are multiple pathways by which uncontained excreta regularly discharged from nearby toilets may pose a risk to young children in urban environments.^16^

Onsite containment of feces, if properly desludged and transported away after filling, is expected to yield lower levels of environmental contamination, and subsequently lower prevalence of enteric infection. Children in these households had the lowest prevalence of enteric infection in this study when compared with those in households without toilets or with toilets but with poor FSM. This finding suggests that fecal contamination—and therefore exposures in the local environment—were lower, though significant differences in drain concentrations were not observed. Though there is systematic evidence showing that sewerage interventions, another form of contained FSM, are associated with decreased incidence of diarrhea and enteric infection, onsite containment with emptying and transport has not been studied.^19^ Further, in contrast to our findings, recent city-level assessments of FSM have shown that long-term management of onsite fecal sludge is generally poor and may lead to nonfunctional toilets and backflow of excreta into homes during floods, contaminating the household environment.^7^,^17^ Given that we did not have the ability to assess the frequency or quality of FSM longitudinally, we cannot definitively make conclusions about the long-term effectiveness of the onsite containment in place. This is an important knowledge gap for future sanitation and health studies, given the increasing frequency with which onsite excreta containment with trucking is observed in urban areas and the prohibitive costs, planning, and operation and maintenance logistics associated with sewer systems.^53^

In areas of low coverage of household toilets, their presence was associated with increased fecal contamination on household floors, which may reflect sharing of toilets and subsequently poorer maintenance and hygiene. Although our study did not collect information on households' sharing of toilets, the practice is not uncommon among households without sanitation in poor urban areas of India.^54^ A recent study of peri-urban homes in Peru indicated household shared sanitation was associated with increased fecal contamination on kitchen floors when compared with individual household sanitation, and household shared sanitation has previously been linked to increased diarrheal prevalence in children.^55^–^57^ However, though it is assumed that shared sanitation yields higher household-level fecal contamination through poor toilet maintenance, further research is necessary as general toilet maintenance was not measured in our study, and recent evidence has suggested that shared sanitation is associated with lower fecal contamination in the toilet itself.^58^

Associations between household toilet presence and increased fecal contamination in low-coverage areas underscore the importance of sanitation at the community level, in addition to the household level. Evidence suggests sanitation coverage—including household toilets and FSM—must surpass a threshold before community-level fecal contamination is sufficiently reduced to confer health benefits.^16^,^22^,^59^ Notably, in previous studies of high coverage of rural sanitation, these health benefits were not limited to only those households with toilets.^23^,^24^,^60^

Although this study included more detailed sanitation exposures, including multiple FSM typologies and spatial heterogeneity, it is limited in its assessment of some potential confounders, including hygiene practices and exposure to animals. Classification of households into “good” or “poor” hygiene based on a 50% score cutoff may have misclassified those with scores around the cutoff. Further, the household hygiene score included questions about practices at the household toilet, limiting our ability to separate conclusions about sanitation and hygiene practices since households without a toilet were more likely to have lower scores. Further, questions about interaction with animals were not part of the household hygiene score or the household survey, thus the contributions of animals to the spread of fecal contamination were not measured.

While a census was not feasible, household selection approximated the spatial distribution of households by random selection within each of five sub-neighborhood areas. Using this approach and estimating clusters by spatial scan provided a more accurate assessment of the spatial heterogeneity underlying the neighborhood, which is significant with regard to socioeconomic variables in low-income, urban settings.^61^

The number of environmental samples and stool specimens collected limited the study's ability to conduct analyses on further subgroups of sanitation within neighborhoods. However, assessment of fecal indicator bacteria and pathogens in environmental samples and enteric infection in stool provided more comprehensive and objective outcomes than previous measures of self-reported diarrhea.^12^,^30^ While detection of pathogens was possible in all cases, inconsistent standard curves for EAEC in drain samples prevented estimation of the CE quantities present, limiting power to detect differences. Although the low prevalence of pathogens in samples within the household limited analyses of *E. coli*, this difference in detection between organisms is important when considering use of fecal indicator bacteria against use of fecal pathogens.

Consideration of sanitation beyond the household toilet, including the household's FSM within the neighborhood environment, is necessary to better understand how reductions in pediatric enteric infection may be achieved in urban areas. Despite new efforts to diagnose FSM conditions, the effects of FSM typologies on fecal contamination and enteric infection are not well described to date, and open drains persist as default sewerage options throughout many low-income countries.^7^,^17^,^62^ At a minimum, future studies should quantify the effects of this hazard at the household, neighborhood, and city scales.^7^

Overall, this study provides evidence of the importance of both FSM and the spatial heterogeneity of household toilets within neighborhoods when evaluating the effectiveness of urban sanitation. To reduce fecal contamination and improve health, good FSM must accompany increases in toilet coverage to remove and safely contain sewage from the urban public and private domains. Because isolated toilets in low-coverage areas may not yield the same benefits as clusters of toilets in high-coverage areas, sanitation must be considered at the community level. As urban populations continue to grow, sanitation interventions must contain excreta in both the public and private domain to protect health and the environment.

## Supplementary Material

Supplemental Datas.

## Figures and Tables

**Table 1:** Reported frequency and clustering of household sanitation and FSM in Chinnallapuram and Old Town

	Chinnallapuram (*N* = 100) Count (%)	Old Town (*N* = 100) Count (%)	Overall (*N* = 200) Count (%)	*P* value*
Household-level				
Household toilet†	78 (78.0)	33 (33.0)‡	111 (55.5)	< 0.01
FSM: Toilet excreta contained onsite§	37 (47.4)	3 (9.1)	40 (36.0)	< 0.01
FSM: Toilet discharges directly to drain§	27 (34.6)	27 (81.8)	54 (48.6)	< 0.01
FSM: Other/do not know§	14 (18.0)	2 (6.1)	16 (14.4)	0.18
Open defecation				
< 5-year-olds	40 (40.0)	80 (80.0)	120 (60.0)	< 0.01
Respondent (adult)	19 (19.0)	68 (68.0)	87 (43.5)	< 0.01
Public toilet use (by respondent)				
None	41 (41.0)	46 (46.0)	87 (43.5)	0.57
Low (1–5 times per month)	51 (51.0)	31 (31.0)	82 (41.0)	0.01
Medium (6–10 times per month)	4 (4.0)	5 (5.0)	9 (4.5)	> 0.99
High (> 10 times per month)	4 (4.0)	18 (18.0)	22 (11.0)	< 0.01
	Chinnallapuram		Old Town	
	Count (cluster prevalence)	*P* value∥	Count (cluster prevalence)	*P* value∥
Most likely clusters¶				
Household toilet				
High-coverage cluster	43 (100.0)	< 0.01	–	–
Low-coverage cluster	40 (50.0)	< 0.01	27 (0.0)	0.02
FSM: toilet discharges directly to drain				
High-coverage cluster	18 (77.8)	0.01	9 (100.0), 7 (100.0)	0.02, 0.04

FSM = fecal sludge management.

* *P* value for *t* test of proportions between neighborhoods.

† All toilets were pour-flush toilets.

‡ Of the 33 households reporting having a toilet, 32 responded to the subsequent questions about FSM.

§ Percent in parentheses represents the percentage of all households with toilets.

¶ No significant clusters of households with toilet excreta contained onsite (good FSM) were observed.

∥ *P* value for comparison of the prevalence of the attribute within the cluster compared with the overall prevalence of the attribute in the neighborhood. Only clusters significant at the 0.05 level are presented, otherwise “–” is presented.

**Table 2:** Variation in detection and concentrations of *Escherichia coli* in environmental samples within households with neighborhood and hygiene status

	Child hand rinse (*N* = 50)				Sentinel object rinses (*N* = 49)*				Household swabs (*N* = 50)†			
	*E. coli* detection	*E. coli* concentration			*E. coli* detection	*E. coli* concentration			*E. coli* detection	*E. coli* concentration		
	Number of samples	Geometric mean (SD)‡			Number of samples	Geometric mean (SD)§			Number of samples	Geometric mean (SD)¶		
Overall	45/50	107.2 (11.7)			32/49	13.2 (5.9)			48/50	245.5 (9.8)		
Associations with neighborhood and hygiene status												
	OR (95% CI)∥	β**	SE(β)	*P* value	OR (95% CI)∥	β††	SE(β)	*P* value	OR (95% CI)∥	β‡‡	SE(β)	*P* value
Neighborhood§§	0.22 (0.01, 1.62)	0.51	0.30	0.09	1.27 (0.39, 4.22)	0.15	0.22	0.51	1.00 (0.04, 26.3)	0.07	0.28	0.81
Poor hygiene¶¶	0.18 (0.01, 1.36)	−0.07	0.31	0.83	0.99 (0.30, 3.28)	0.19	0.22	0.40	0.85 (0.03, 22.2)	−0.28	0.28	0.33
Neighborhood§§	0.19 (0.01, 1.48)	0.51	0.30	0.10	1.28 (0.39, 4.23)	0.16	0.22	0.48	0.99 (0.04, 26.1)	0.06	0.28	0.84
Poor hygiene¶¶	0.16 (0.01, 1.24)	−0.05	0.30	0.88	1.01 (0.31, 3.34)	0.20	0.22	0.38	0.85 (0.03, 22.3)	−0.28	0.28	0.34

CI = confidence interval; OR = odds ratio; SD = standard deviation; SE = standard error.

* One sentinel object rinse was unable to be read and thus was not included in the results. Sentinel objects were plastic (28/50), metal (17/50), other material (4/50), or mixed material (1/50).

† Household floors were cement (39/50), tile (8/50), or other material (3/50).

‡ Units are colony-forming unit (CFU)/pair of hands.

§ Units are CFU/100 mL.

¶ Units are CFU/125 cm^2^.

∥ Though *P* values for ORs are omitted for reasons of space in the table, none were significant at α = 0.05.

** Estimate is in log_10_ CFU/pair of hands.

†† Estimate is in log_10_ CFU/100 mL.

‡‡ Estimate is in log_10_ CFU/125 cm^2^.

§§ Old Town neighborhood (reference is Chinnallapuram).

¶¶ Hygiene status was divided into “poor” or “good” hygiene categories based on a 18-point scale (0–9 as “poor,” 10–18 as “good”) discussed in Methods section and presented in Collinet-Adler and others.^34^

**Table 3:** Variation in detection of enteric pathogens in stool with neighborhood and hygiene status*

	Any enteric pathogen		*Campylobacter* spp.		*Giardia* spp.		Pathogenic *Escherichia coli*†	
	PR (95% CI)	*P* value	PR (95% CI)	*P* value	PR (95% CI)	*P* value	PR (95% CI)	*P* value
Neighborhood: Old Town	1.32 (0.50, 3.49)	0.57	1.91 (0.71, 5.14)	0.18	1.56 (0.48, 6.46)	0.45	0.73 (0.14, 3.78)	0.64
Poor hygiene	1.97 (0.75, 5.62)	0.17	3.42 (1.30, 12.3)	0.02	1.69, (0.53, 7.27)	0.39	0.55 (0.07, 3.37)	0.34
Neighborhood: Old Town	1.37 (0.51, 3.72)	0.53	2.15 (0.80, 5.97)	0.13	1.60 (0.49, 6.78)	0.47	0.70 (0.11, 5.02)	0.56
Poor hygiene	2.01 (0.76, 5.77)	0.16	3.61 (1.37, 11.4)†	0.01	1.72 (0.54, 7.57)	0.54	0.53 (0.09, 4.10)	0.31

CI = confidence interval; PR = prevalence ratio for detection of enteric pathogen in stool specimen.

* *N* = 76 children from which stool specimens were collected (43 in Chinnallapuram, 33 in Old Town). Enteric pathogens detected in stool specimens included astrovirus, *Campylobacter* spp., *Entamoeba histolytica*, *Giardia* spp., genotype II norovirus, and pathogenic *E. coli*. A full list of organisms tested in stool specimens is presented in Houpt and others.^29^ Only pathogens detected in > 20% of stool specimens were regressed against neighborhood and hygiene status.

† Enteroaggregative *E. coli*, enterohemorrhagic *E. coli*, enteropathogenic *E. coli*, and enterotoxigenic *E. coli*.

**Table 4:** Variation in GII norovirus detection and concentration in drain water

	GII norovirus detection		GII norovirus concentration		
	OR (95% CI)*	*P* value	β†	SE(β)	*P* value
Demographics and hygiene					
Neighborhood: Old Town	0.42 (0.13, 1.34)	0.15	−0.38	0.39	0.35
Poor hygiene	0.92 (0.29, 2.91)	0.88	−0.05	0.40	0.90
Neighborhood: Old Town	0.42 (0.12, 1.33)	0.15	−0.38	0.40	0.35
Poor hygiene	0.88 (0.27, 2.86)	0.83	−0.07	0.41	0.87
Sanitation (household level)					
Household toilet	4.73 (0.93, 28.9)	0.07	0.90	0.53	0.10
Toilet excreta contained onsite‡	1.51 (0.37, 6.10)	0.56	0.41	0.49	0.41
Toilet discharges to drain‡	1.14 (0.28, 4.74)	0.85	0.17	0.50	0.74
Toilet excreta contained onsite§	1.81 (0.38, 8.95)	0.45	0.61	0.55	0.28
Toilet discharges to drain§	1.50 (0.31, 7.62)	0.61	0.44	0.55	0.43
Open defecation (< 5-year-old)	0.07 (0.01, 0.58)	0.02	−0.95	0.52	0.07
Open defecation (< 5-year-old)/OT	0.69 (0.05, 9.67)¶	0.09¶	NI∥	–	–
Open defecation (adult)	0.08 (< 0.01, 0.78)	0.05	−0.62	0.58	0.30
Open defecation (adult)/OT	1.10 (0.05, 22.5)¶	0.09¶	NI∥	–	–
Any public toilet use (adult)	1.47 (0.44, 4.96)	0.53	0.33	0.42	0.43
High public toilet use (> 10 times per month, adult)	2.38 (0.36, 17.0)	0.36	0.75	0.64	0.24
Sanitation (cluster level)					
High HH toilet coverage	3.32 (0.62, 21.2)	0.17	1.47	0.57	0.01
Low HH toilet coverage	1.05 (0.30, 3.68)	0.94	−0.51	0.43	0.24
High coverage of poor FSM	2.29 (0.31, 17.8)	0.41	2.50	1.04	0.02
High coverage of poor FSM/OT	NI∥	–	−2.34	1.26	0.07

CI = confidence interval; FSM = fecal sludge management; GII = genotype II; HH = household; NI = no interaction; OR = odds ratio; OT = Old Town; SE = standard error.

* Models are adjusted for neighborhood and hygiene status (“good” or “poor”, as discussed previously). Interactions of sanitation variable and neighborhood were tested for all models and are presented if *P* < 0.10.

† Concentration differences are in log_10_ genome equivalent copies/100 mL.

‡ Estimated relative to all other households, including those with toilets with other associated FSM and those without toilets.

§ Estimated relative to households without a toilet or those with “other” FSM practices.

¶ OR for interaction is presented as predicted OR for the OT neighborhood (from the model), not as the exponentiation of the interaction term alone. *P* value presented is for the interaction term alone.

∥ NI with neighborhood was included in this model (*P* ≥ 0.10 for interaction term).

**Table 5:** Any enteric pathogen detection in child stool* by household- and cluster-level attributes†

	PR (95% CI)	*P* value
Household level		
Household toilet	1.57 (0.45, 5.49)	0.48
Toilet excreta contained onsite‡	0.45 (0.14, 1.43)	0.17
Toilet discharges to drain‡	10.0 (1.52, 200)¶	0.04¶
Toilet discharges to drain/OT	0.58 (0.04, 7.94)¶	0.03¶
Toilet excreta contained onsite§	0.69 (0.12, 3.73)	0.67
Toilet discharges to drain§	8.33 (1.02, 181)	0.08
Toilet excreta contained onsite/OT	0.66 (0.05, 8.62)¶	0.98¶
Toilet discharges to drain/OT	0.53 (0.03, 8.61)¶	0.05¶
Open defecation (< 5-year-old)	0.38 (0.10, 1.50)	0.17
Open defecation (adult)	0.83 (0.21, 3.32)	0.79
Any public toilet use (adult)	1.50 (0.54, 4.20)	0.44
High public toilet use (> 10 times per month, adult)	0.78 (0.16, 3.74)	0.76
Cluster level§		
High cluster of household toilets	0.75 (0.17, 3.33)	0.71
Low cluster of household toilets	0.73 (0.26, 2.09)	0.56
High cluster of household toilets discharging to drain	2.55 (0.43, 15.1)	0.30

CI = confidence interval; OT = Old Town; PR = prevalence ratio.

* Pathogens detected in stool included astrovirus, *Campylobacter* spp., *Entamoeba histolytica*, *Giardia* spp., genotype II norovirus, and pathogenic *Escherichia coli*.

† Models with PRs for detection of any enteric pathogen in stool specimens presented, adjusted for neighborhood and hygiene status (“good” or “poor”).

‡ Estimated relative to all other households, including those with toilets with other associated fecal sludge management (FSM) and those without a toilet.

§ Estimated relative to households without a toilet or those with “other” FSM practices.

¶ Odds ratio (OR) for interaction is presented as predicted OR for the OT neighborhood (from the model), not as the exponentiation of the interaction term alone. *P* value presented is for the interaction term alone.
