# Supplementary material for: The Influence of Household- and Community-Level Sanitation and Fecal Sludge Management on Urban Fecal Contamination in Households and Drains and Enteric Infection in Children
Source: Am J Trop Med Hyg. 2017 Jun 7;96(6):1404–14. doi: 10.4269/ajtmh.16-0170 (PMC5462580; doi:10.4269/ajtmh.16-0170)
Supplement: Supplementary file 1 [file SD6.pdf]

SUPPLEMENTAL INFORMATION

CHRISTIAN MEDICAL COLLEGE HYGIENE SURVEY[28]

Questions	Response	Scoring
1. Do they have a devoted container to store drinking water?	0-no; 1-yes	0-**0**; 1-**1**
2. Is the container covered?	0-no; 1-yes	0-**0**; 1-**1**
3. Is there a dipper dedicated for drinking water?	0-no; 1-yes; 2-dipper not used	(0,2)-**0**; 1-**1**
4. Is the drinking water boiled?*	0-no; 1-yes, for someone; 2- yes for everybody	0-**0**; (1,2)-**1**
5. Is the food kept covered?	0-no; 1-yes	0-**0**; 1-**1**
6. Are the fruits washed before eating?	0-no; 1-yes	0-**0**; 1-**1**
7. Cleaning the breast before feeding	0-no; 1-yes; 9-NA	(0,9)-**0**; 1-**1**
8. Are the nails cut properly/without dirt in between the nails?	0-no; 1-yes	0-**0**; 1-**1**
9. Washing the hands before feeding	0-no; 1-yes	0-**0**; 1-**1**
10. Feeding device washed properly	0-no; 1-yes, by soap; 2-yes, by water; 9- NA	(0,9)-**0**; 1-**1**; 2-**2**
11. Water available in the toilet*	0-no tap; 1-tap present	0-**0**; 1-**1**
12. Handwashing after going to toilet*	0-no; 1-water only; 2- water and soap	0-**0**; 1-**1**; 2-**2**
13. Periodicity of bathing*	0-not daily; 1-once daily; 2- twice daily	0-**0**; (1,2)-**1**
14. Bathing soon after going to toilet*	0-no; 1-yes	0-**0**; 1-**1**
15. Place of defecation of household*	1-household; 2-public; 3-field; 4-backyard/surroundings	(3,4)-**0**; (1,2)-**1**
16. Where does the child defecate?*	1-household; 2-public; 3-field; 4-backyard/surroundings	(3,4)-**0**; (1,2)-**1**

Total number of criteria = **16**. All the category scores were added up to obtain a final hygiene score. Maximum score = **18**. All questions carried **1** score for positive response except "feeding device washed properly" and "handwashing after going to toilet" score **1** for washing with "water alone" and **2** for washing with "water and soap." Bolded values represent the score assigned for a given question.

*These questions were asked to the respondent whereas the others were observations.

STOOL SPECIMEN PROCESSING

Collected specimens were analyzed for bacteria, helminths, protozoa, and viruses. Bacteria were identified using a variety of methods. Culture-based methods were used for the isolation and identification of *Salmonella*, *Shigella*, *Aeromonas*, *Vibrio*, *Plesiomonas*, *Yersinia*, and *Escherichia coli*, and *E. coli* isolates were further identified as pathogenic *E. coli* using PCR-based assays that were designed to detect Shiga toxin–producing *E. coli*, enterotoxigenic *E. coli*, enteropathogenic *E. coli*, enteroinvasive *E. coli*, and enteroaggregative *E. coli* (EAEC).[29] *Campylobacter* spp. were also analyzed using polymerase chain reaction (PCR)–based methods.[30] Protozoa (*Entamoeba histolytica*, *Cryptosporidium*, and *Giardia* spp.) and viruses (adenovirus, astrovirus, and rotavirus) were evaluated by enzyme-linked immunosorbent assays, with the exception of norovirus, which was identified by reverse transcription PCR. Other ova and parasites, including soil-transmitted helminths, were identified by morphology using a wet prep and modified acid-fast stain.[29]

QUANTITATIVE REAL-TIME PCR

Total nucleic acids were extracted using the Qiagen Xtractor system (Qiagen Sciences, Germantown, MD), following manufacturer's instructions. EAEC was detected using primers and probes targeting the *aatA* gene.[31] GI norovirus was detected using genogroup-specific COG1 primers and RING1-TP probe, while GII norovirus was detected using the genogroup-specific COG2 primers and RING2-TP probe.[32] The standard curve for EAEC was generated from a plasmid containing the *aatA* gene. The standard curve for GI and GII noroviruses was generated from in vitro transcribed RNA.[33]

SUPPLEMENTAL TABLE 1

Analysis of *Escherichia coli* concentrations in child hand rinses by household- and cluster-level sanitation and FSM*

	β†	Standard error	*P* value
Household-level			
Household toilet	0.04	0.40	0.92
Toilet contains excreta onsite‡	0.04	0.41	0.92
Toilet discharges to drain‡	0.56	0.41	0.17
Open defecation (< 5-year-old)	0.52	0.39	0.19
Open defecation (adult)	0.34	0.43	0.44
Any public toilet use (adult)	−0.04	0.31	0.91
High public toilet use (> 10 times per month, adult)	−0.01	0.48	0.99
Most likely clusters			
High household toilet coverage	−0.44	0.44	0.32
Low household toilet coverage	−0.13	0.32	0.69
High coverage of poor FSM	0.05	0.51	0.93

*Multivariate models are presented for each sanitation variable, adjusting for neighborhood and hygiene status ("poor" or "good", as discussed previously), and are separated by a blank row in the table. In all models, interaction terms between the sanitation variable and neighborhood were tested and are indicated with a "/" if significant at $\alpha = 0.10$, and $N = 50$ samples.

†Estimates are in $\log_{10}$ colony-forming units (CFU)/pair of hands.

‡Reference population is households without a toilet or those with "other" FSM practices.

Supplemental Table 2

Analysis of *Escherichia coli* concentrations in household swabs by household- and cluster-level sanitation and FSM*

	β†	Standard error	*P* value
Household level			
Household toilet	–0.14	0.49	0.78
Household toilet/Old Town	1.15	0.59	0.06
Toilet contains excreta onsite‡	0.33	0.38	0.39
Toilet discharges to drain‡	0.57	0.38	0.15
Open defecation (< 5-year-old)	0.24	0.37	0.52
Open defecation (adult)	0.10	0.49	0.84
Open defecation (adult)/neighborhood: Old Town	–1.15	0.59	0.06
Any public toilet use (adult)	0.41	0.29	0.16
High public toilet use (> 10 times per month, adult)	0.52	0.45	0.25
Most likely clusters			
High household toilet coverage	–0.27	0.42	0.52
Low household toilet coverage	0.43	0.39	0.27
Low cluster of household toilet coverage/Old Town	–1.38	0.56	0.02
High coverage of poor FSM	0.42	0.48	0.39

FSM = fecal sludge management.

*Multivariate models are presented for each sanitation variable, adjusting for neighborhood and hygiene status ("poor" or "good", as discussed previously), and are separated by a blank row in the table. In all models, interaction terms between the sanitation variable and neighborhood were tested and are indicated with a "/" if significant at $\alpha = 0.10$, and $N = 50$ samples.

†Estimates are in $\log_{10}$ colony-forming units (CFU)/125 cm^2.

‡Reference population is households without a toilet or those with "other" FSM practices.

Supplemental Table 3

Analysis of *Escherichia coli* concentrations in sentinel object rinses by household- and cluster-level sanitation and FSM*

	β†	Standard error	*P* value
Household level			
Household toilet	0.06	0.30	0.83
Toilet contains excreta onsite‡	0.03	0.30	0.91
Toilet discharges to drain‡	0.16	0.30	0.60
Open defecation (< 5-year-old)	0.37	0.29	0.20
Open defecation (adult)	–0.14	0.32	0.66
Any public toilet use (adult)	–0.14	0.23	0.54
High public toilet use (> 10 times per month, adult)	–0.08	0.35	0.82
Cluster level			
High household toilet coverage	–0.08	0.33	0.80
Low household toilet coverage	–0.01	0.24	0.97
High coverage of poor FSM	–0.14	0.37	0.71

FSM = fecal sludge management.

*Multivariate models are presented for each sanitation variable, adjusting for neighborhood and hygiene status ("poor" or "good", as discussed previously), and are separated by a blank row in the table. In all models, interaction terms between the sanitation variable and neighborhood were tested and are indicated with a "/" if significant at $\alpha = 0.10$, and $N = 49$ samples.

†Estimates are in $\log_{10}$ colony-forming units (CFU)/100 mL.

‡Reference population is households without a toilet or those with "other" FSM practices.
